# Induction of apoptosis in experimental human B cell lymphomas by conditional TRAIL-expressing T cells

**DOI:** 10.1038/sj.bjc.6601407

**Published:** 2003-11-25

**Authors:** E Ucur, J Mattern, T Wenger, S Okouoyo, A Schroth, K-M Debatin, I Herr

**Affiliations:** 1 Clinical Cooperation Unit, Molecular Oncology/Pediatrics, German Cancer Research Center, Heidelberg, Germany; 2 University Children's Hospital, Ulm, Germany; 3 Clinical Cooperation Unit Oncological Diagnostics and Therapy, German Cancer Research Center, Heidelberg, Germany

**Keywords:** TRAIL, gene therapy, Tet system, apoptosis, B cell lymphoma

## Abstract

In the present study, we demonstrate the utility of a non-tumour-forming T-cell line for the inducible gene transfer of tumour necrosis factor (TNF)-related apoptosis-inducing ligand (Apo2L/TRAIL), which has been shown to selectively induce apoptosis in malignant but not in normal cells. To generate T cells inducible for TRAIL expression, we stably transfected Jurkat cells with TRAIL in the context of the Tet-On system. The switched on cells strongly expressed TRAIL mRNA, whose protein product was expressed on the cell surface. Paracrine induction of apoptosis in human target tumour cells was solely found for membrane-bound TRAIL. The Jurkat-TRAIL cells itself survived due to clonal selection of TRAIL-resistant cells. Jurkat-TRAIL cells had an additive effect with cytotoxic drugs *in vitro*, since cell death was enhanced. To elucidate the antitumoral activity of these Jurkat-TRAIL cells *in vivo*, we injected them intratumorally in xenografts of human Burkitt lymphomas. Switching on expression of TRAIL by adding tetracycline to the drinking water of the mice strongly reduced tumour growth by apoptosis in a caspase-dependent manner. Thus, non-tumour-forming T-cell lines offer a novel method for gene transfer and inducible expression of TRAIL in tumour therapy.

Tumour necrosis factor (TNF)-related apoptosis-inducing ligand (Apo2L/TRAIL) is a member of the TNF family of cytokines. The apoptosis-inducing receptors for TRAIL include TRAIL-R1 (DR4) and TRAIL-R2 (DR5), which are expressed on the surface of many types of cells. TRAIL-R1- or -R2-mediated apoptosis requires the FADD adaptor molecule, and leads to the activation of the initiator caspase-8, which activates downstream caspases (Walczak and Krammer, 2000).

TRAIL is suggested to induce apoptosis preferentially in a wide range of transformed cell lines, while most normal cells were resistant both *in vitro* (Wiley *et al*, 1995; Pitti *et al*, 1996) and *in vivo* (Walczak *et al*, 1997; Ashkenazi *et al*, 1999). In contrast, injection of other death ligands such as CD95-L or TNF-*α* results in massive degeneration of normal tissue (Ashkenazi *et al*, 1999). The tumour selectivity and safety of soluble recombinant TRAIL *in vivo* have been examined in various studies, and it was generally found to be well tolerated even when multiple doses were administered to animals (Ashkenazi *et al*, 1999; Walczak *et al*, 1999; Fulda *et al*, 2002). Furthermore, tumoricidal activity of TRAIL *in vivo* is enhanced when combined with chemotherapeutic agents, ionising radiation or Smac peptides (Nagane *et al*, 2001; Fulda *et al*, 2002; Munshi *et al*, 2002). Surprisingly, previous investigators have reported the toxic effects of TRAIL also on normal primary human cells, including hepatocytes (Jo *et al*, 2000), keratinocytes (Leverkus *et al*, 2003) and endothelial cells (Li *et al*, 2003). The basis of this difference is unclear, but could result from the methods used to assess cell death or problems in preparation of the respective recombinant TRAIL used in these particular studies (Lawrence *et al*, 2001). Also, primary keratinocytes are relatively resistant and become first sensitive by inhibition of the proteasome (Leverkus *et al*, 2003). In this regard, the effects of TRAIL-induced injury in primary endothelial cells are not unique. The observation of Li *et al* (2003) that primary endothelial cells are susceptible to TRAIL death signals differ from those reported previously by others (Ashkenazi *et al*, 1999; Walczak *et al*, 1999). However, although Li *et al* (2003) found that TRAIL, compared to TNF, is potent at causing injury, it was less effective at stimulating inflammation in endothelial cells. Therefore, the transfer of recombinant TRAIL protein remains a promising antitumour agent.

In the present study, the non-tumour-forming leukaemic T-cell line Jurkat was used as vehicle for a new and alternative approach to transfer TRAIL under the control of a tetracyclin (tet)-regulated promoter to tumour cells. Upon switching on the expression of TRAIL, these manipulated cells induced strong paracrine apoptosis in human Burkitt lymphoma (BJAB) cells *in vitro* and *in vivo.* The antitumoral effect of TRAIL was specifically mediated by membrane-bound TRAIL via the death receptor pathway, and enhanced the therapeutic potential of cytotoxic drugs.

## RESULTS

### Tet-inducible RNA and cell surface protein expression of TRAIL in switched on Jurkat-TRAIL cells

For inducible expression of TRAIL, we chose the Tet-On system, which consists of a transactivator (rtTA) and an expression (pTRE) construct. In the presence of tet, the tetracycline-controlled reverse transactivator protein (rtTA) expressed by pTet-On binds to its target site within the pTRE promoter, and drives the expression of the respective downstream gene.

The full-length cDNA of human TRAIL was cloned into the pTRE expression plasmid and cotransfected into Jurkat cells stably expressing rtTA resulting in tet-inducible Jurkat-TRAIL cells. As a control, the Jurkat-rtTA cells were transfected with the empty pTRE plasmid (Jurkat-CO). After selection for hygromycin B resistance, we cultivated the cells in increasing concentrations of tet for several days to induce clonal selection of TRAIL-resistant cells. Resistance of Jurkat-TRAIL cells towards TRAIL is a necessary prerequisite for TRAIL donor cells. Otherwise, sensitive Jurkat-TRAIL cells would undergo apoptosis upon TRAIL expression and could not be used as vehicle for the transfer of TRAIL.

**Figure 1:**
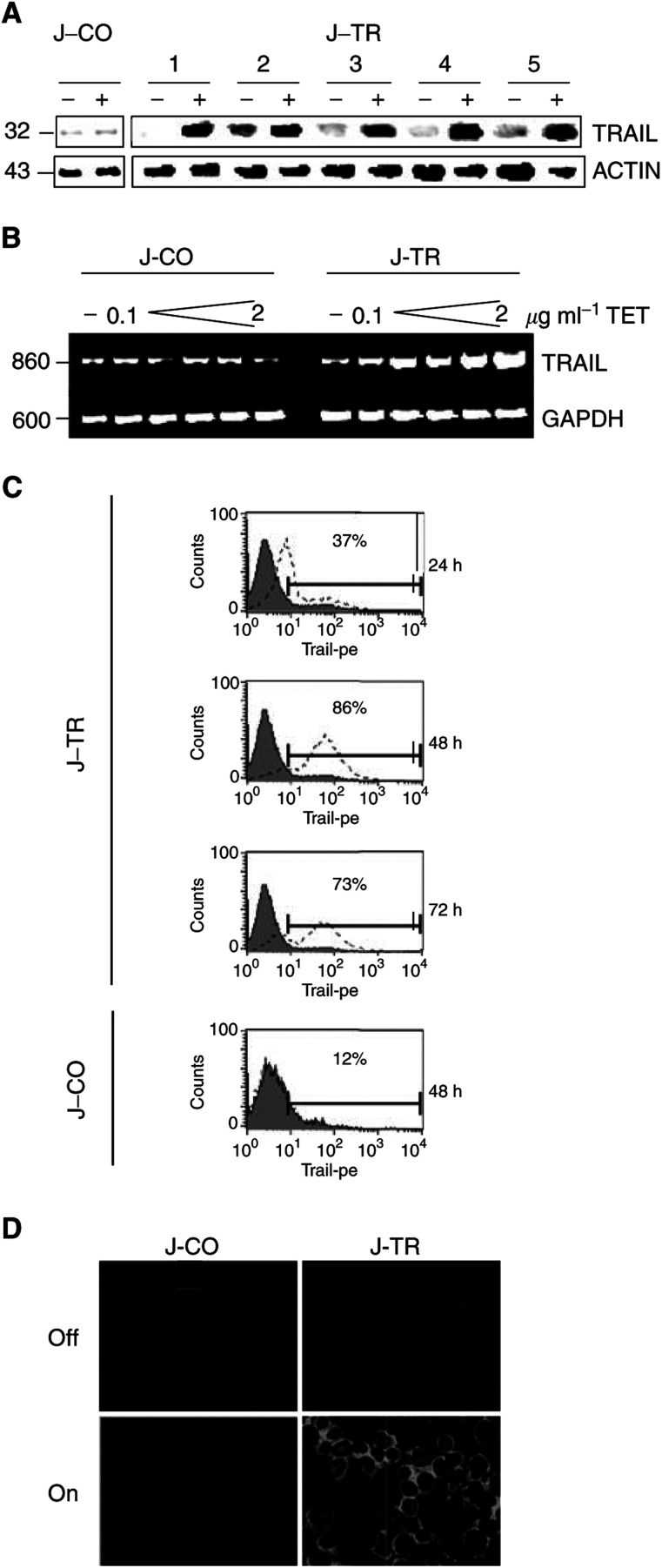
Tet induces TRAIL overexpression in Jurkat-TR (J-TR) cells. (**A**) Five selected single clones of J-TR or one clone of Jurkat-CO cells (J-CO) were cultured for 48 h in the presence (+, switched on) or absence (−, switched off) of tet (2 *μ*g ml^−1^). Proteins were extracted and TRAIL expression was assayed by Western blot analysis using a mouse mAb, which specifically detects the 32 kDa TRAIL protein. Expression of the 43 kDa *α*-ACTIN protein was examined as a control for equal conditions. (**B**) Jurkat-TRAIL (J-TR) clone 5 and Jurkat-CO (J-CO) cells were incubated in the presence (+) or absence (−) of increasing doses of tet (0, 0.1, 0.5, 1, 1.5, 2 *μ*g ml^−1^). After 48 h, total RNA was harvested and RT–PCR was performed using TRAIL-specific primers. Assaying the RNA expression of GAPDH confirmed equal conditions. (**C**) Jurkat-TRAIL (J-TR) or Jurkat-CO (J-CO) cells were cultured in the presence (open histograms with dotted lines) or absence (filled histograms with solid line) of tet (2 *μ*g ml^−1^) and analysed by flow cytometry. The number indicates the percentage of TRAIL-positive cells. (**D**) Jurkat-TRAIL (J-TR) clone 5 and Jurkat-CO cells were switched on (2 *μ*g ml^−1^ tet) or left switched off. After 48 h, cytospins were prepared and the cell surface expression of TRAIL was analysed by immunofluorescence staining. The representative results shown are from one of three different experiments with similar outcomes.

Surviving single clones were assayed for inducible protein expression of TRAIL. Five clones out of 112 Jurkat-TRAIL cells in the switched on status showed strong upregulation of TRAIL, in contrast to Jurkat-CO cells in which no induction was observed (Figure 1A

). For dose–response assays, we treated Jurkat-TRAIL and Jurkat-CO cells with increasing concentrations of tet, and analysed the RNA expression of TRAIL by RT–PCR. Tet was used in the range of 0.5–2 *μ*g ml^−1^, since these are recommended optimum concentrations for inducible gene expression by the Tet-On system. While only a minimal response was observed with a tet concentration of 0.5 *μ*g ml^−1^, maximal expression of TRAIL by Jurkat-TRAIL was reached at 2 *μ*g ml^−1^ (Figure 1B). In contrast, endogenous TRAIL expression in Jurkat-CO cells was unchanged. In order to quantify cell surface expression of TRAIL, we performed flow cytometry analysis of switched on Jurkat-TRAIL cells. After 24 h, 37% of the cells showed strong TRAIL cell surface expression. After 48 h, the number of TRAIL-positive cells reached a peak of 86%, which dropped back to 73% after 72 h (Figure 1C). In Jurkat-CO cells, TRAIL expression remained at a low level. To further highlight these results, we analysed cell surface expression by immunofluorescence staining using an alternative specific antibody against human TRAIL (Figure 1D). In line with the flow cytometry data, strong membrane expression of TRAIL protein was detected in switched on Jurkat-TRAIL cells only.

### Induction of apoptosis in human Burkitt lymphoma target cells by Jurkat-TRAIL cells

**Figure 2:**
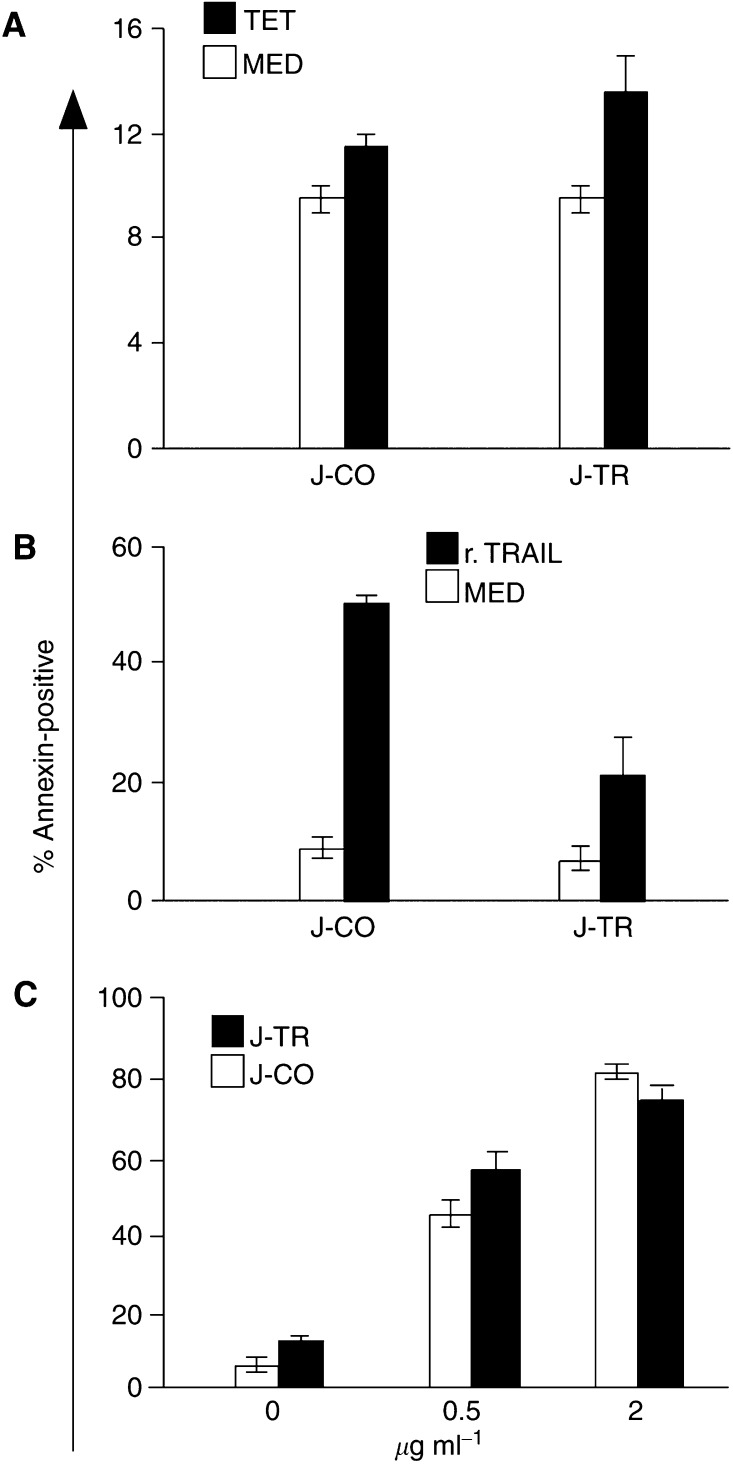
Jurkat-TRAIL cells are resistant towards TRAIL, but not cisplatin. (**A**) Jurkat-TRAIL (J-TR) and Jurkat-CO (J-CO) cells were switched on (2 *μ*g ml^−1^ tet) (black bars) or left switched off (white bars). After 48 h, apoptosis was measured by staining of the cells with annexin/PI and FACS analysis. (**B**) Switched off Jurkat-TRAIL and Jurkat-CO cells were treated with 100 ng ml^−1^ human recombinant TRAIL protein (black bars) or left untreated (white bars), and apoptosis was determined. (**C**) Switched off Jurkat-CO (white bars) and Jurkat-TRAIL (black bars) were treated with 0.5 or 2 *μ*g ml^−1^ cisplatin, as indicated. After 48 h, apoptosis was determined. Data are presented as means of triplicate samples and s.d. is shown.

Due to the selection conditions, we expected that the Jurkat-TRAIL cells itself are resistant towards TRAIL-induced apoptosis. To examine this point, we switched on the system and measured cell death by staining of the cells with annexin/FITC, followed by flow cytometry analysis (Figure 2A

). At 48 h after induction, no significant percentage of apoptosis was detectable, since the level of apoptosis in switched on Jurkat-TRAIL cells resembled basal death in switched off Jurkat-CO cells. Also, exogenously added recombinant soluble TRAIL protein induced a minor percentage of 21% apoptosis in Jurkat-TRAIL cells, but a level of 50% in Jurkat-CO cells (Figure 2B). Thus, Jurkat-TRAIL cells exhibit resistance towards TRAIL-induced apoptosis, which may result from clonal selection. To test whether Jurkat-TRAIL cells still remained sensitive to other death-inducing agents, we treated them with the chemotherapeutic agent cisplatin. Afte 48 h, we found strongly elevated levels of apoptosis, similar to the levels obtained with Jurkat-CO cells. Therefore, Jurkat-TRAIL cells are selectively resistant towards TRAIL (Figure 2C).

**Figure 3:**
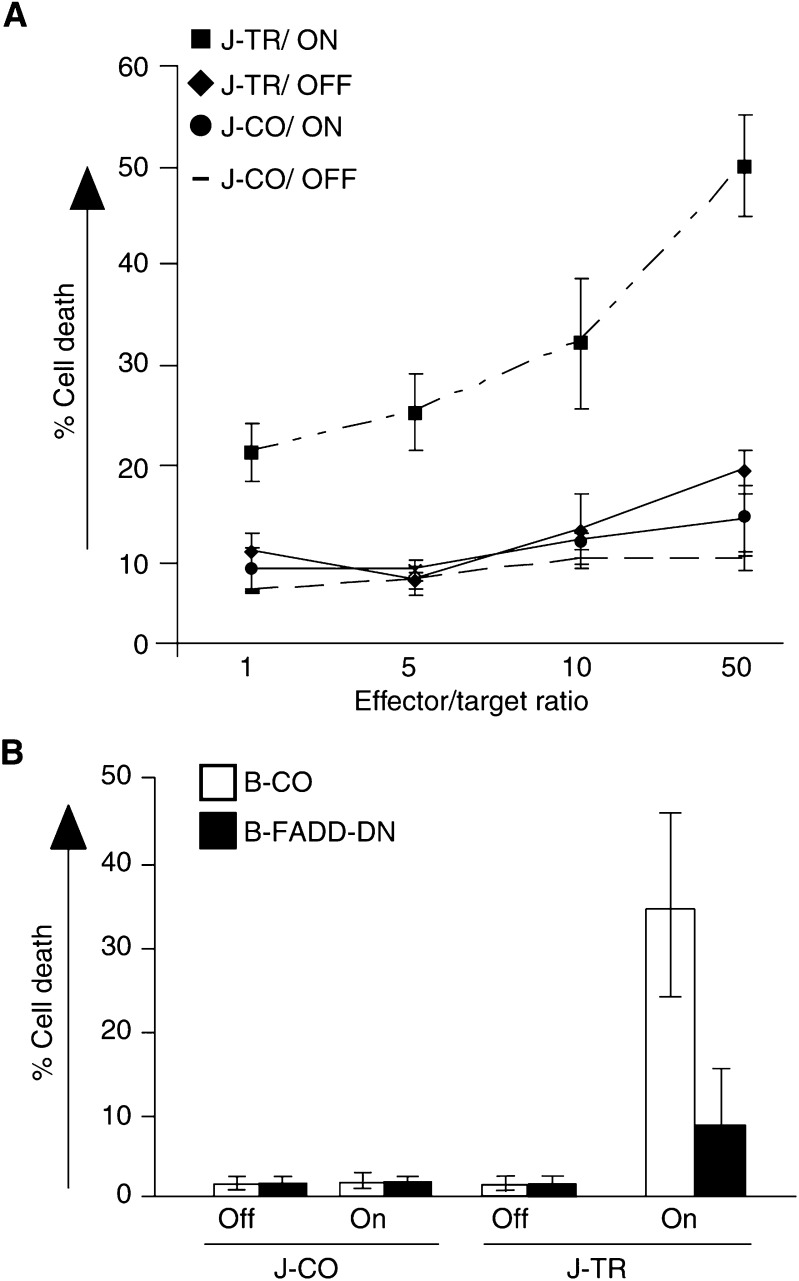
Jurkat-TRAIL cells mediate paracrine death. (**A**) Jurkat-TRAIL (J-TR) and Jurkat-CO (J-CO) cells were switched on with tet (2 *μ*g ml^−1^) and added at the indicated ratios to BJAB cells stably transfected with GFP by lentiviral-mediated gene transfer. Cell death was measured by flow cytometry using FSC/SSC and gating on green fluorescent BJAB cells. The percentage of specific death was calculated as follows: 100 × (experimental death (%)−spontaneous death in the control (%)]/[100%−spontaneous death in the control (%)). (**B**) Jurkat-TRAIL and Jurkat-CO cells, either switched on or not, were incubated with BJAB cells stably transfected with dominant-negative FADD (black bars) or with empty pcDNA3 vector (white bars), at an *e*/*t* of 50 : 1. BJAB cells were stained with an FITC-coupled antibody directed towards the B-cell-specific cell surface receptor CD19, and cell death was measured by flow cytometry using FSC/SSC and gating on green fluorescent BJAB cells. After 48 h, the percentage of specific death was determined as described above. Data are presented as means of triplicate samples and s.d. is shown. The representative results shown are from one of three different experiments.

To investigate the cytotoxicity of Jurkat-TRAIL cells, we performed a donor/target kill assay and used GFP-expressing BJAB cells as targets. Jurkat-TRAIL or Jurkat-CO cells were switched on with 2 *μ*g ml^−1^ tet, followed by co-culturing with GFP-expressing BJAB cells at increasing effector/target (*e*/*t*) ratios from 1 : 1 up to 50 : 1. At 24 h after incubation, cell death was measured via gating on green-fluorescent BJAB cells by flow cytometry (Figure 3A

). Death started to elevate in BJAB cells co-cultured with Jurkat-TRAIL cells at an *e*/*t* of 1 : 1, and increased with higher ratios. No significant induction of apoptosis was observed in BJAB cells co-cultured with Jurkat-CO or with switched-off Jurkat-TRAIL cells, at any *e*/*t* used. Also, co-culturing of BJAB cells with the supernatant from switched on Jurkat-TRAIL cells did not result in induction of cell death at any ratio, as detected visually or by Annexin-V staining up to 72 h post-treatment (data not shown). These data indicate that death was mediated solely by membrane-bound TRAIL and not by the shedded soluble death ligand. Next, we looked whether paracrine death might be specifically mediated by the death receptor pathway rather than by any cytotoxic side effect. We used BJAB cells with a blocked death receptor signalling due to the stable expression of dominant-negative FADD (BJAB-FADD-DN). BJAB cells expressing empty pcDNA3 vector (BJAB-CO) were used as control. At an *e*/*t* of 50 : 1, Jurkat-TRAIL or Jurkat-CO cells were co-cultured with BJAB-FADD-DN or with the BJAB-CO cells. After 48 h, death was observed in BJAB-CO only, but not in BJAB-FADD-DN cells, although both cell lines have been incubated with switched on Jurkat-TRAIL cells. Together, upon switching on, membrane-bound TRAIL of Jurkat-TRAIL cells specifically induce paracrine death via the death receptor pathway in TRAIL-sensitive tumour target cells *in vitro*.

### Jurkat-TRAIL cells enhance chemotherapy-induced apoptosis in BJAB target cells

**Figure 4:**
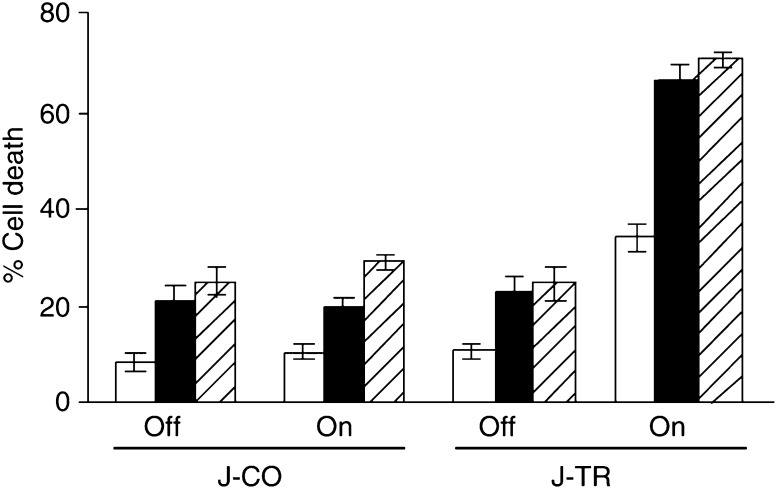
Jurkat-TRAIL cells enhance chemotherapy-induced apoptosis of target cells. BJAB cells were pretreated with cisplatin (1 *μ*g ml^−1^, solid black bars), doxorubicin (100 ng ml^−1^, striped bars) for 6 h or left untreated (white bars). Jurkat-TRAIL (J-TR) or Jurkat-CO (J-CO) cells, switched on (2 *μ*g ml^−1^ tet, 48 h) or not, were added at an *e*/*t* of 50 : 1 to the pretreated BJAB cells. After 24 h, the percentage of cell death in BJAB cells was determined by flow cytometry, as described in Figure 3B. Data are presented as means of triplicates and s.d. is shown. The representative results shown are from one of three different experiments.

Since TRAIL-induced apoptosis has been shown to be augmented by cotreatment with cytotoxic drugs (Nagane *et al*, 2001; Munshi *et al*, 2002), we investigated whether tet-induced TRAIL protein expression would enhance chemotherapy-mediated apoptosis in our system. BJAB cells were pretreated with cisplatin or doxorubicin for 6 h and then co-cultured with Jurkat-TRAIL or Jurkat-CO cells in the switched on or off status. After 24 h, cell death was measured in BJAB cells. While cisplatin or doxorubicin alone induced between 20 and 30% death, cytotoxicity was dramatically increased to 70% by co-culturing switched on Jurkat-TRAIL cells (Figure 4

). In contrast, the presence of Jurkat-CO cells in any status or switched off Jurkat-TRAIL cells did not further enhance drug-induced apoptosis. These data demonstrate that paracrine death induced by switched on Jurkat-TRAIL cells strongly increases chemotherapy-induced apoptosis in sensitive tumour cells.

### Jurkat-TRAIL cells inhibit tumour growth of TRAIL-sensitive human xenografts

**Figure 5:**
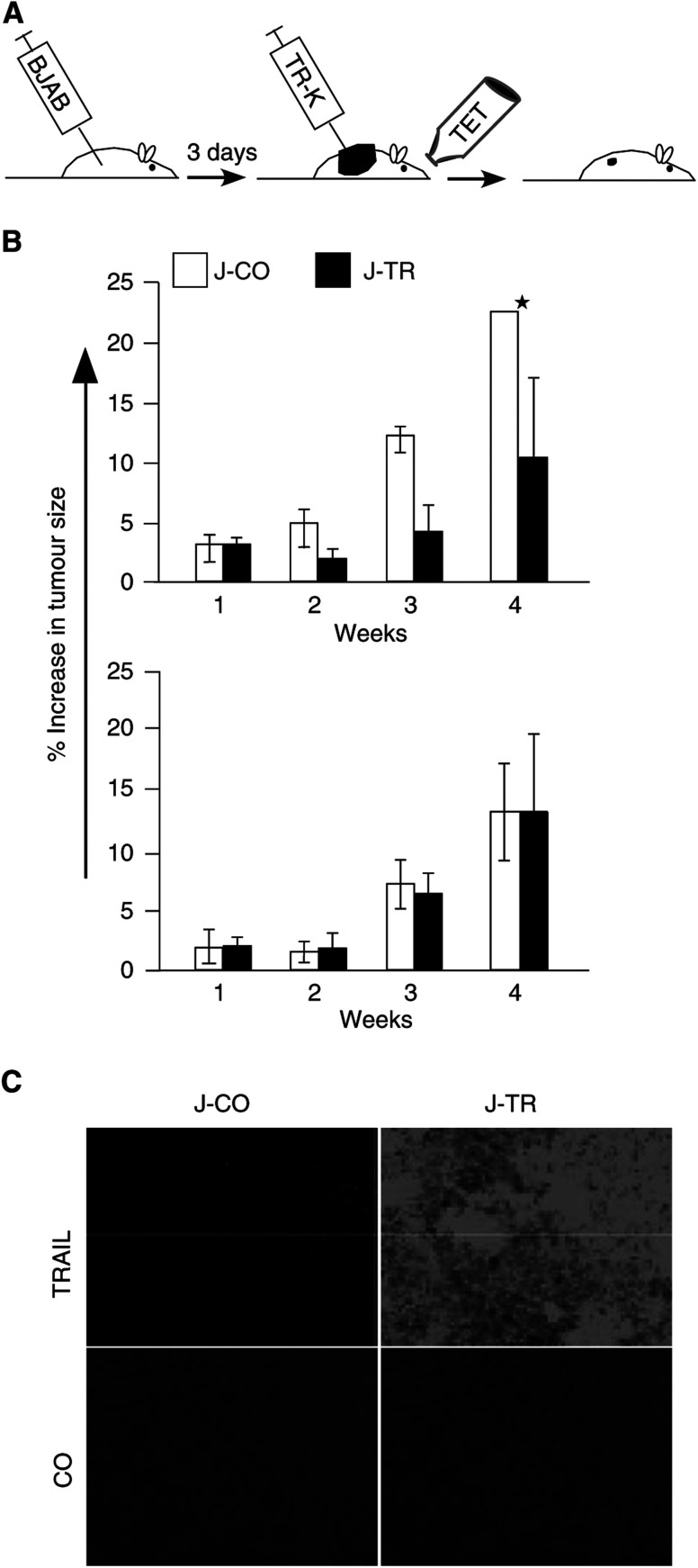
Jurkat-TRAIL cells inhibit tumour growth of xenografted BJAB cells. (**A**) Schematic representation of the experiment. Athymic nude mice were xenografted with 5 × 10^7^ BJAB cells. After 3 days, 5 × 10^7^ Jurkat-TRAIL or Jurkat-CO cells were injected intratumorally. To switch on the Tet-system, tet (1 mg ml^−1^) was added to the drinking water, which was sweatened with 5% glucose for the duration of the experiment. (**B**) Upper panel: tumour growth of BJAB xenografts, injected with switched on Jurkat-TRAIL (black bars) or Jurkat-CO cells, was weekly measured during a period of 4 weeks. Data are presented as the means of eight animals and s.d. are shown (^*^=13.08). Lower panel: nude mice were xenografted with 5 × 10^7^ Kelly cells and treated as described above. Data are presented as the means of four animals and s.d. is shown. (**C**) Cryosections of BJAB xenografts harvested 3 days upon switching on TRAIL expression were stained with a specific rabbit polyclonal anti-TRAIL antibody, followed by immunofluorescence detection. Staining or the absence of the primary antibody served as control (CO). The representative results shown are from one of three different experiments.

**Figure 6:**
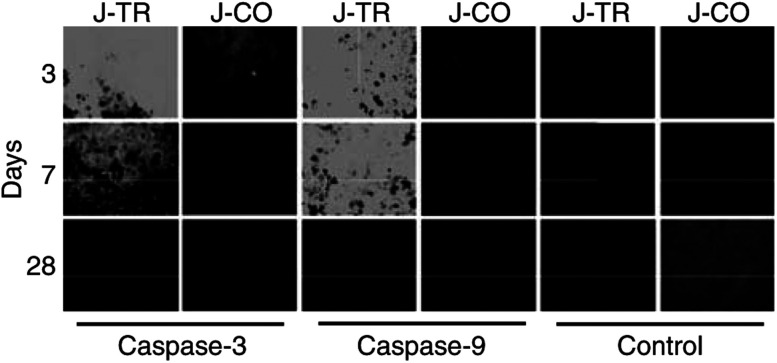
Jurkat-TRAIL cells induce caspase activity *in vivo*. Nude mice were treated as described in Figure 5. BJAB xenografts were harvested at the indicated time points. Cryosections were incubated with polyclonal rabbit caspase-9 or -3 antibodies, both of which specifically detect the cleaved active caspase fragments. The bound antibodies were detected with fluorescein-conjugated anti-rabbit IgG. Rabbit IgG directed towards goat IgG's served as negative control. The representative results shown are from one of three different experiments.

Since Jurkat cells itself do not expand and do not form tumours in mice (unpublished observation), we assessed the effect of Jurkat-TRAIL cells in an *in vivo* tumour model. Athymic nude mice were xenografted with BJAB cells. After 3 days, at tumour volumes of about 100 mm^3^, animals were injected intratumorally with switched off Jurkat-TRAIL or Jurkat-CO cells. Tet was added to the drinking water of the mice to switch on the system (Figure 5A

). Weekly measurement of the tumour volume over a period of 4 weeks revealed a profound reduction of tumour growth in animals inocculated with switched on Jurkat-TRAIL cells (Figure 5B). In contrast, BJAB xenografts inocculated with Jurkat-CO cells increased continuously and growth was not affected by the presence of tet in the drinking water. Next, we tested whether Jurkat-TRAIL cells may specifically induce apoptosis via the death receptor pathway *in vivo*. However, for this experiment, we could not use BJAB-FADD-DN, since this cell line does not form tumours in nude mice. Therefore, we used apoptosis-resistant neuroblastoma (Kelly) cells defective in death receptor signalling due to a hypermethylated and downregulated caspase-8 gene (Teitz *et al*, 2000; Fulda *et al*, 2001). Kelly xenografts were treated as described above. As expected, no decrease in tumour growth occurred, demonstrating that reduction of B-cell lymphomas by Jurkat-TRAIL is not due to any unspecific toxic side effects, but critically depends on intact death receptor signalling (Figure 5B, lower panel). In control experiments, we examined the expression of TRAIL in BJAB xenografts by immunofluorescence analysis. At 3 days after injection of effector cells, cryosections were prepared and stained with a specific TRAIL antibody. High fluorescence was detected in BJAB xenografts inocculated with the switched on Jurkat-TRAIL cells only (Figure 5C). To further investigate whether the delay of tumour growth was specifically due to the induction of apoptosis, we analysed the activity of caspase-3 and -9 by immunofluorescence staining (Figure 6

). Tumour sections obtained 3, 7 or 28 days after injection of effector cells were stained with anti-caspase-9 or -3 antibodies, specific for the cleaved and active caspase fragments only. The highest fluorescence-reflecting caspase activity was detected in BJAB xenografts injected with switched on Jurkat-TRAIL cells 3 days after injection. Although caspase activity dropped back, the signal was still pronounced at day 7, but completely absent at day 28, suggesting that Jurkat-TRAIL cells have been eliminated at this time point. Therefore, switched on Jurkat-TRAIL cells profoundly reduce the growth of human BJAB xenografts.

### Jurkat-TRAIL cells reduce the growth of large tumours

**Figure 7:**
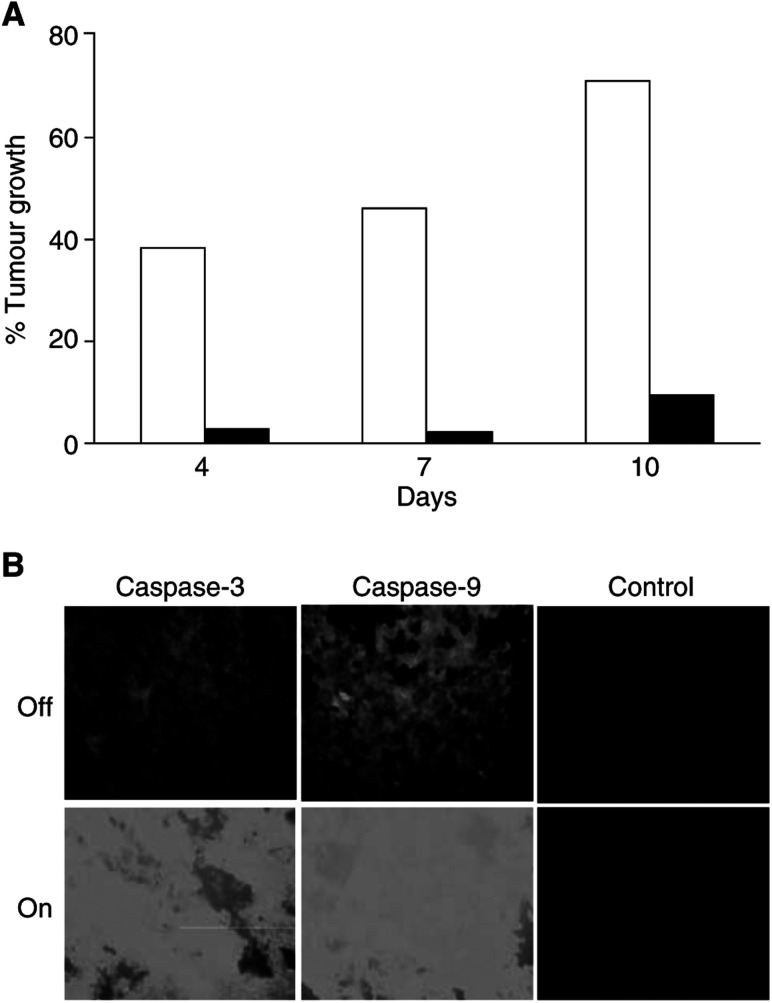
Jurkat-TRAIL cells reduce the growth of large tumours. (**A**) Nude mice were xenografted with BJAB cells, as described in Figure 5. After 2 weeks, tumours reached a size of about 1000 mm^3^, and were inocculated with Jurkat-TRAIL cells. Likewise, mice received tet (1 mg ml^−1^) in the glucose-sweetened drinking water (black bars) or not (white bars). Injection of Jurkat-TRAIL cells was repeated 3 days later. Tumour growth was measured 4, 7 and 10 days after the first injection. (**B**) Caspase activity was detected at day 10 after the first injection, as described in Figure 6. The representative results shown are from one of three different experiments with similar results.

Next, we investigated, whether Jurkat-TRAIL cells may also be able to reduce tumour growth of larger xenografts. BJAB xenografts with an average size of 1000 mm^3^ were inoculated with Jurkat-TRAIL cells (Figure 7

). Since we already showed in Figure 6 that Jurkat-Co cells did not influence tumour growth, we used now switched off Jurkat-TRAIL cells as control. Therefore, to switch on the system, half of the mice received tet-containing drinking water, whereas control mice received water without tet. The injection of effector cells was repeated 3 days after the first injection of Jurkat-TRAIL cells to enhance the effect. Tumour growth was monitored 4, 7 and 10 days after the first treatment. Whereas switched on Jurkat-TRAIL cells elucidated a substantial delay in tumour growth, the growth of B-cell lymphomas inocculated with switched off Jurkat-TRAIL cells was seven-fold higher. Finally, to confirm the specific tumour reduction by TRAIL-induced apoptosis, we detected the activity of caspase-3 and -9 in xenografts 10 days after the first treatment. Intense fluorescence was observed in tumours of animals, which received switched on Jurkat-TRAIL cells only. Thus, apoptosis mediated by tet-induced TRAIL-expression induces a delay in tumour growth also in advanced tumour stages.

## DISCUSSION

The present study describes a novel anticancer gene therapeutic approach, using a non-tumour-forming T-cell line, which was generated to conditionally express TRAIL on the cell surface. We chose the widely used model of Jurkat cells since we found that these leukaemia cells itself do not expand or form solid tumours upon injection in mice, and may be therefore suited for *in vivo* therapeutical studies. Out of the TNF superfamily of death-inducing ligands, TRAIL was taken since it is described to selectively induce apoptosis in a large variety of cancer cells but not in normal cells (Ashkenazi *et al*, 1999; Walczak *et al*, 1999). This feature makes TRAIL a potential antitumour agent. In an attempt to combine selectivity with regulated gene expression, we stably cloned the human TRAIL cDNA in the context of the Tet-On system. This system is most suited, since previous studies demonstrated that tet-regulated overexpression of apoptotic genes mediates cell death in transfected cancer cells. Thus, tet-induced overexpression of BAX enhanced apoptosis induced by chemotherapeutic agents (Kobayashi *et al*, 2002). Similarly, Faris *et al* (1998) utilised the Tet-Off system for overexpression of a dominant-active MEKK1 molecule, which mediated apoptosis by upregulation of CD95-L. In the present study, we engineered Tet-On Jurkat-TRAIL cells, which exhibit strong overexpression of TRAIL mRNA and cell surface protein upon induction. However, in contrast to parental Jurkat cells, Jurkat-TRAIL cells do not undergo apoptosis in response to overexpressed TRAIL due to clonal selection of TRAIL-resistant cells. Selection of TRAIL-resistant cells by the continuous high expression of TRAIL is not unique to our system, since a recent report also describes the selection of TRAIL-resistant Jurkat subclones after continuous treatment with exogenously added recombinant TRAIL protein (Kim *et al*, 2001). Switched on Jurkat-TRAIL cells strongly induced paracrine death in target human Burkitt lymphoma BJAB cells. Death was specifically mediated by membrane-bound TRAIL, since we could not detect the induction of paracrine death by the supernatant of tet-treated Jurkat-TRAIL cells. This is another benefit of our system, compared to direct administration of soluble recombinant TRAIL in multiple other studies (Ashkenazi *et al*, 1999; Walczak *et al*, 1999; Fulda *et al*, 2002). Paracrine induction of death by Jurkat-TRAIL is restricted to the immediate surrounding area of neighbouring cells, thereby avoiding systemic side effects, since soluble active TRAIL may induce hepatotoxicity when released in the circulation *in vivo*. Induction of apoptosis was specifically mediated by overexpressed TRAIL, since it was only observed by using switched on Jurkat-TRAIL but not by Jurkat-CO cells. In addition, apoptosis of target tumour cells in our system depends on intact death receptor pathways and is not mediated by any unspecific cytotoxic side effects. Accordingly, BJAB target cells with a blocked death receptor pathway due to the expression of dominant-negative FADD did not undergo apoptosis upon paracrine expression of TRAIL. Also, xenografts of neuroblastoma cells with a defective caspase-8 gene (Teitz *et al*, 2000; Fulda *et al*, 2001) did not respond to TRAIL.

An additional potential advantage of our gene therapy approach is the inducible sustained expression and antitumour activity of TRAIL, as well as the expression in its natural context as a membrane protein. To this end, switched on Jurkat-TRAIL cells caused a continuous wave of cell death in human tumour xenografts, as compared to recombinant TRAIL that only leads to a short-term burst of apoptosis. The former mechanism might be of therapeutic benefit, since it could inhibit the generation of TRAIL-resistant cells due to the persistent presence of overexpressed TRAIL.

Viral delivery systems for TRAIL have been recently reported, for example, using adenoviral (Griffith and Broghammer, 2001) or AAV vectors (Mohr *et al*, 2003). However, whereas AAV vectors may provide a relatively safe *in vivo* delivery method for therapeutic genes, recombinant adenovirus may induce a severe immune response in patients. Furthermore, severe induction of apoptosis in primary human hepatocytes was reported by adenoviral gene transfer of TRAIL (Armeanu *et al*, 2003), which further restricts the use of this delivery method.

In contrast to previous studies (Walczak *et al*, 1999; Griffith and Broghammer, 2001; Kagawa *et al*, 2001), our approach reduces also the growth of larger tumours, since in these other studies therapy was started prior to the development of a tumour. Thus, TRAIL was injected in parallel with the xenografted tumour cells. Also, Griffith and Broghammer (2001) reported that adenoviral-expressed TRAIL exhibited the best antitumoral effect, when injected just 1 day after tumour implantation. To mimic more closely the situation in patients, we delivered Jurkat-TRAIL cells when the tumours reached sizes of already 100 up to 1000 mm^3^, and found a reduction in growth of Burkitt lymphomas in either case.

A somehow controversial point of view is the proposed selectivity of TRAIL, which suggests induction of apoptosis only in malignant but not in normal cells (Ashkenazi *et al*, 1999; Walczak *et al*, 1999). In this context, cultured human hepatocytes or human primary epithelial cells were recently reported to undergo apoptosis in response to recombinant TRAIL, raising concerns of the potential toxicity of TRAIL to normal tissue *in vivo* (Jo *et al*, 2000; Nesterov *et al*, 2002). However, this inconvenience may be attributed to problems in preparation of the respective recombinant TRAIL used in these particular studies (Lawrence *et al*, 2001), since multiple other reports including our study did not detect the unspecific toxic side effects *in vivo* (Griffith and Broghammer, 2001; Kagawa *et al*, 2001; Mohr *et al*, 2003). Thus, mice receiving switched on Jurkat-TRAIL cells looked healthy, behaved normal and survived due to the benefit of reduced tumour growth.

One more advantage of our system is due to the observed strong additive effect of switched on Jurkat-TRAIL cells with chemotherapeutic agents, which markedly enhanced the toxicity of cisplatin and doxorubicin in BJAB cells, similar to other reports describing a synergistic action of recombinant TRAIL protein with cytotoxic drugs and *γ*-irradiation (Bonavida *et al*, 1999; Keane *et al*, 1999). Thus, pretreatment, especially of therapy-resistant tumours with inducible TRAIL donor cells, may sensitise them for chemotherapy-induced apoptosis, which finally may also eliminate the TRAIL-producing donor cells.

In conclusion, this study is the first to show inducible long-term expression of nonviral-delivered TRAIL in xenografted human tumours with a single intratumoral injection of donor cells. The apoptosis-inducing activity of membrane-bound TRAIL is restricted to neighbouring cells, thereby achieving a restricted bystander effect in order to avoid side effects on normal tissue. Combination of inducible TRAIL expressed by non-tumour-forming T cells with conventional therapy might further increase the efficacy of cancer treatment especially in resistant tumours, which might be sensitised by pretreatment with TRAIL.

## MATERIAL AND METHODS

### Cell culture

Parental Jurkat (human T-cell leukaemia line), Jurkat-derived subclones Jurkat-CO (stably transfected with the reverse transactivator rtTA and an empty response plasmid pTRE) or Jurkat-TRAIL (stably transfected with rtTA and a pTRE-TRAIL expression plasmid), BJAB (human Burkitt lymphoma cell line), either wildtype or stably transfected with a FADD-dominant negative construct or empty pcDNA3 vector and neuroblastoma Kelly cells (kindly provided by Dr M Schwab), were grown at 37°C in RPMI supplemented with 10% foetal bovine serum, 1% HEPES, 1% glutamine (all from Life Technologies, Gibco, Karlsruhe, Germany) and 2.5 *μ*g ml^−1^ plasmocin (*In vivo*Gen, San Diego, USA). Transfected cells were cultivated in the presence of 400 *μ*g ml^−1^ G418 (Life Technologies) or 400 *μ*g ml^−1^ hygromycine B (Roche, Mannheim, Germany). Mycoplasma contamination of all cell lines was excluded by the VenorGem test (Minerva Biolabs, Berlin, Germany), following the manufacturer's instructions.

### Lentiviral infection of BJAB cells with GFP

BJAB cells stably expressing green fluorescent protein (GFP) were engineered by infection with a HIV-1-based GFP-expressing lentiviral vector, as described (Wenger and Herr, unpublished).

### Nude mice and xenografts

BJAB cells (5 × 10^7^ cells in 200 *μ*l PBS) were injected s.c. into the subaxillary region of 6–10-weeks-old BALBc (nu/nu) female mice, which were maintained by conventional methods. To switch on the expression of TRAIL, the mice were given 1 mg ml^−1^ tet (doxycycline, Clontech, Heidelberg, Germany) in drinking water, and the amount of water consumed was monitored. Effector cells were injected intratumorally. The tumour growth was followed by measuring two diameters with callipers. The tumour volume was calculated by the formula *V*=(*a*^2^ × *b*)/2, where is *a* the width and *b* the length in mm. All the experiments have been carried out with ethical committee approval, and meet the standards required by the UKCCCR guidelines (Workman *et al*, 1998).

### Stimulation of cells

Cisplatin (Sigma, Deisenhofen, Germany) was dissolved in DMSO at a concentration of 10 *μ*g *μ*l^−1^, which was stored in aliquots at −80°C. Doxorubicin (Sigma, Deisenhofen, Germany) was diluted in 70% EtOH to a concentration of 100 ng *μ*l^−1^, and stored in aliquots at −80°C. Recombinant human soluble TRAIL/APO2L (Peprotech, London, England) was diluted in water to a concentration of 0.1 *μ*g *μ*l^−1^ and stored in aliquots at −20°C.

### Cloning of TRAIL cDNA

The full-length cDNA was cloned by RT–PCR from Jurkat cell RNA, using primers described (Wiley *et al*, 1995). The oligos carried additional restriction enzyme sites (5′ *Eco*RI, 3′ *Bam*HI) in order to facilitate cloning into pTRE Clontech (Heidelberg, Germany). The resulting vector was checked by sequence analysis.

### Tet-on system and stable transfectants

The Tet-on system consisting of Jurkat cells stably transfected with Tet-induced regulator plasmid pTet-On (Jurkat-Tet-On) and the response plasmid pTRE were obtained from Clontech (Heidelberg, Germany). Inducibility of Jurkat-Tet-On was confirmed by transient transfection of a pTRE luciferase construct. The TRAIL cDNA was cloned in pTRE using the *Eco*RI and *Bam*HI restriction sites. The resulting pTRE-TRAIL plasmid was cotransfected in the Jurkat-Tet-On cell line. In detail, 5 × 10^8^ Jurkat cells in 200 *μ*l PBS were cotransfected with 40 *μ*g pTRE-TRAIL, or with an empty pTRE vector in the presence of 2 *μ*g pTK-Hyg by electroporation (975 *μ*F, 220 V). After transfection, the cells were resuspended in 12 ml fresh medium. After 48 h, the cells were washed in PBS and a selection medium containing hygromycine B was added and the cells were plated to six-well test plates, followed by selection for 8 weeks.

### Western blot analysis

Protein expression was detected by Western blotting, as described (Herr *et al*, 2003). Mouse mAb anti-TRAIL was obtained from BD-Pharmingen (Heidelberg, Germany). As a control for equal protein-loading, membranes were restained with mAb *α*-ACTIN (ICN, Eschwege, Germany). Bound antibodies were detected by anti-mouse/horseradish peroxidase conjugates (Santa Cruz, Heidelberg, Germany) and enhanced chemiluminescence.

### RT–PCR

Total RNA was harvested, converted to cDNA and PCR was performed as previously described (Herr *et al*, 2003). The following primer sequences were used: human TRAIL: gcaggaattcaggatcatggctatgatgg and gcacggatcccaggtcagttagccaact (860 bp), GAPDH: ccacccatggcaaatttctccatggca and tctagacggcaggtcaggtccacc (600 bp).

### Quantitation of cell surface expression of TRAIL

Cells were incubated with polyclonal rabbit antibody against human TRAIL (Santa Cruz Biotechnology, California, USA), diluted 1 : 80 in PBS containing 1% FCS, followed by biotinylated secondary goat anti-rabbit (KPL, Guildford, UK), and streptavidin-PE (BD-Pharmingen, Heidelberg, Germany). Surface staining was determined on a FACScalibur (Becton Dickinson, Heidelberg, Germany).

### Measurement of apoptosis

Early apoptotic changes were identified by staining of cells with fluorescein thiocyanate (FITC)-conjugated annexin V and propidium iodide (Becton Dickinson, Heidelberg, Germany), and analysed by flow cytometry (FACScan, Becton Dickinson), as described (Herr *et al*, 2003).

### Cytoxicity assay

Target BJAB cells were transduced with a GFP-expressing lentiviral vector (BJAB-H/CiGW) at an MOI of 5. Expression of GFP and sensitivity of the transduced cells towards soluble recombinant TRAIL was ensured by flow cytometric analysis. Jurkat cells were cultivated in cell culture medium containing 2 *μ*g ml^−1^ tet (doxycycline) for 48 h to induce TRAIL expression. Thereafter, the cells were washed twice with the media, and combined at 4 × 10^5^ (*e*/*t* ratio 10 : 1) or 2 × 10^6^ (*e*/*t* ratio 50 : 1) with 4 × 10^4^ target BJAB cells in 200 *μ*l well^−1^ of a 96-well tissue culture plate. Plates were centrifuged for 2 min at 800 × **g** to ensure the close contact of effector and target cells. After cultivation at 37°C for 24 h, cell death in BJAB-H/CIGW cells was determined by FACS analysis (FACScan BD, Heidelberg, Germany) using FSC/SSC and gating on the green fluorescent BJAB cells. BJAB-FADD-DN cells were cultivated at 37°C for 48 h and stained with an FITC-labelled antibody directed towards the B-cell-specific cell surface receptor CD19 (BD-Pharmingen, Heidelberg, Germany), before performing FACS analysis.

### Immunofluorescence staining of cells

Cytospins were prepared and fixed in 100% methanol. Unspecific binding was reduced by incubation in 10% Roti®-Immunoblock (Roth, Karlsruhe, Germany). Expression of TRAIL was detected by a mouse mAb anti-human TRAIL (BD/Pharmingen), diluted 1 : 250 in PBS-Tween containing 1.5% Roti®-Immunoblock. The bound antibodies were detected by FITC-conjugated anti-mouse IgG (Molecular Probes Europe, Leiden, The Netherlands). Cells were mounted in KAISER's glycerol gelatine (MERCK, Darmstadt, Germany) and examined by fluorescence microscopy.

### Immunofluorescence staining of tissue sections

Frozen xenograft sections were air dried and fixed in 100% methanol. Unspecific binding was reduced by incubation in 10% Roti®-Immunoblock (Roth, Karlsruhe, Germany). Activity of caspase-9 and -3 was detected using polyclonal rabbit caspase-9 (Asp353, from Cell Signalling Technology, Frankfurt, Germany), or caspase-3 (BD/Pharmingen, Heidelberg) antibodies raised against the active subunits. The bound antibodies were detected by fluorescein-conjugated goat anti-rabbit IgG (Molecular Probes, OR, USA). TRAIL expression was detected with rabbit polyclonal anti-TRAIL antibody (Santa Cruz Biotechnology, CA, USA) and Alexa Fluor 488 conjugated secondary anti-rabbit antibody (MoBiTec, Göttingen, Germany). To ensure the specificity of the immunostaining reactions, consecutive tissue sections were incubated in the absence of the primary antibody or with rabbit IgG (Dako, Glostrup, Denmark) directed towards goat IgG's.
